# Clinical Patterns of Osteoarticular, Bone Marrow and Urogenital Paracoccidioidomycosis

**DOI:** 10.1111/myc.70182

**Published:** 2026-04-28

**Authors:** Wdson Luis Lima Kruschewsky, Mariane Cappellini Stricagnolo, Mônica Scarpelli Martinelli Vidal, Gil Benard, Vítor Falcão de Oliveira, Tiago Alexandre Cocio, Adriana Satie Gonçalves Kono Magri, Marcello Mihailenko Chaves Magri

**Affiliations:** ^1^ Infectious Diseases and Tropical Medicine Department Hospital das Clínicas da Faculdade de Medicina da Universidade de São Paulo São Paulo Brazil; ^2^ Faculty of Medicine of the Municipal University of São Caetano do Sul São Paulo Brazil; ^3^ Laboratory of Medical Mycology ‐ LIM‐53, Institute of Tropical Medicine, Division of Dermatology, Hospital das Clínicas, Faculdade de Medicina da Universidade de São Paulo São Paulo Brazil; ^4^ Instituto do Câncer do Estado de São Paulo, Faculdade de Medicina da Universidade de São Paulo São Paulo Brazil

**Keywords:** atypical, bone marrow, osteoarticular, Paracoccidioidomycosis, rare forms, urogenital

## Abstract

**Background:**

Paracoccidioidomycosis (PCM) is an endemic systemic mycosis in Latin America, classically characterized by pulmonary, mucosal and lymphatic involvement. Extrapulmonary manifestations affecting uncommon organs remain poorly characterized and may contribute to diagnostic delay and adverse outcomes.

**Methods:**

We conducted a retrospective case series of proven or probable atypical extrapulmonary PCM diagnosed at a Brazilian tertiary referral center between 2006 and 2025. A systematic review registered in PROSPERO and conducted according to PRISMA recommendations identified osteoarticular, bone marrow and urogenital PCM cases between 1970 to November 2025 in PubMed/MEDLINE and Embase without language restrictions.

**Results:**

Among 205 PCM patients evaluated at our institution, 14 (6.8%) presented atypical extrapulmonary involvement. Combined with 153 cases identified in 75 studies, 167 cases were analysed. Osteoarticular involvement was the most frequent manifestation (53.9%), predominantly affecting young patients with acute/subacute disease and presenting mainly as osteomyelitis with lytic lesions. Bone marrow involvement occurred exclusively in acute/subacute PCM and was associated with post‐mortem diagnosis (9.7%) and markedly elevated mortality (77.8%). Urogenital PCM mainly affected middle‐aged men with chronic disease, frequently involving the testicles and prostate, and was associated with delayed diagnosis, surgical intervention (32.7%), and mortality (32.0%). Distinct epidemiological profiles, clinical forms and outcomes were consistently observed across atypical sites.

**Conclusion:**

Atypical extrapulmonary PCM manifestations follow recognizable clinical patterns associated with diagnostic delay and poor outcomes, supporting earlier investigation and timely antifungal therapy in endemic settings.

## 1 Introduction

Paracoccidioidomycosis (PCM) is a systemic infection caused by species of the *Paracoccidioides brasiliensis* complex and *Paracoccidioides lutzii*. PCM is a prevalent and endemic infection throughout Central and South America, with around 80% of cases documented in Brazil [1]. Between 1930 and 2012, more than 15,000 cases were registered in Latin America [2]. However, this number only partially illustrates the actual burden of this mycosis. The absence of compulsory notification and underdiagnosis contribute to the hardship in accurately tracking its epidemiology.

Primary infection usually is asymptomatic and normally occurs in the first two decades of life [1]. When it causes disease, PCM can present in two distinct clinical forms. The most common is the chronic form, which typically affects adults between 30 and 60 years of age and is characterized by pulmonary involvement and lesions of the upper aerodigestive tract mucosa [1, 3]. The acute/subacute form is more frequently observed in individuals under 30 years of age and primarily involves the reticuloendothelial system, being characterized by fever, localized or generalized lymphadenopathy and hepatosplenomegaly [1, 3].

PCM infection can be easily mistaken for other diseases due to its capacity to disseminate and involve virtually any organ or system. While typical cases are well documented in the literature, the presentation and characterization of atypical PCM sites remain highly variable, even in endemic regions such as Brazil [3]. This variability can lead to diagnostic delays and contribute to the increased morbidity and mortality associated with the disease [1].

Most descriptions of atypical cases come from case reports with unusual pulmonary and cutaneous presentations [4, 5]. Nonetheless, little is known about the involvement of rare sites by the disease, such as the osteoarticular, bone marrow and urogenital systems. At the same time, there are no studies defining what constitutes atypical or rare site involvement in PCM. Therefore, this study reports a case series of rare PCM sites from a Brazilian reference center and systematically reviews published cases of atypical sites in PCM, emphasizing their main epidemiological and clinical characteristics.

## 2 Methods

This was a retrospective study with secondary data analysis. We included cases of proven or probable PCM at atypical sites, diagnosed at the Hospital das Clínicas, Faculty of Medicine, University of São Paulo (HCFMUSP), between 2006 and 2025. Proven cases are characterized by patients with clinical manifestations compatible with PCM, in whom typical yeast forms of *Paracoccidioides* species have been identified in any clinical sample through direct mycological examination, culture or histopathology. Probable cases are marked by suggestive clinical manifestations and the detection of specific antibodies in serum, but without identification of *Paracoccidioides* species in clinical samples [1]. Atypical forms comprised organ involvements not classically recognized as typical PCM manifestations in current consensus guidelines and major reviews of the disease, where pulmonary, mucosal, lymphatic and cutaneous involvements predominate [1, 3]. Therefore, we defined atypical sites as clinical involvement occurring in fewer than 5% of cases.

Then, a literature review was performed, following the PRISMA guidelines [6], using the databases PubMed/MEDLINE and Embase, which were last consulted in November 2025. Studies were retrieved using the terms described in the Appendix A. We did not use any restrictions regarding languages. Aiming to expand the search, references of the selected articles were reviewed to find additional articles. We registered the protocol on PROSPERO (CRD420261302185). Two qualified investigators (WLLK and MCS) independently assessed the eligibility of the identified publications and discrepancies were resolved by discussion. We first performed an initial screen of titles or abstracts to assess potential relevance. Afterward, we obtained relevant full‐text articles, reevaluated their eligibility and determined their final inclusion or exclusion. Criteria for inclusion were articles: (i) reporting proven or probable PCM; (ii) describing atypical PCM manifestations (osteoarticular, bone marrow and urogenital); (iii) published between 1970 and November 2025. If there was more than one study published using the same case, the most recent study was selected for analysis. We excluded reviews, studies with animals, and in vitro studies.

We employed a standardized pilot‐tested data collection form that comprised the publication year, country, age, sex, occupation, smoking, alcoholism, comorbidities, clinical form (acute/subacute or chronic), clinical manifestations, duration of symptoms, tissue/organ involvement, diagnosis method (direct microscopy, culture, histology and serology), autopsy, hospitalization, antifungal therapy, surgical treatment and outcomes (improvement and death).

We evaluated specific findings regarding the individual patient data collected on atypical forms. In osteoarticular PCM, cases were categorized as arthritis, osteomyelitis, tenosynovitis/bursitis and/or myositis. Arthritis was classified as monoarticular (one joint involved), oligoarticular (two to four joints involved) and polyarticular (more than four joints involved). The other conditions (osteomyelitis, tenosynovitis/bursitis and myositis) were classified as unifocal (a single affected site) and multifocal (more than one affected site). Additional signs and symptoms assessed included local pain, edema, hyperemia and fistula formation. For a more comprehensive assessment, we included magnetic resonance imaging (MRI), computed tomography (CT), ultrasound (USG) and X‐ray. Radiological abnormalities comprised joint effusion, synovitis, abscess, soft tissue edema, lytic lesions and periosteal reaction.

Cases were categorized as genital, renal, or urinary tract involvement in urogenital PCM, with documentation of unilateral or bilateral disease. Signs and symptoms assessed included genital lesions, urgency, nocturia, hematuria, pollakiuria, dysuria, urinary retention, local pain and edema. In cases with bone marrow involvement, the presence of anaemia, pancytopenia and eosinophilia was evaluated.

Simple descriptive statistics, such as the mean and Standard Deviation (SD), frequency and median, were used to characterize the data.

Ethical approval for the study was obtained from the local ethics committee (Approval date: 09/11/2024; approval file: 7000069187). Informed consent was waived because this study was retrospective, with the review of medical records.

## 3 Results

### 3.1 Case Series

A total of 205 patients with proven or probable PCM at HCFMUSP between 2006 and 2025 were analysed, of whom 14 (6.8%) presented with atypical involvement, including osteoarticular, bone marrow and urogenital sites. Of these, 10 (71.4%) had osteoarticular involvement, 3 (21.4%) had bone marrow involvement, and 4 (28.6%) had urogenital involvement, with some patients presenting overlapping involvement across sites. The median age was 40 years (range: 4–66), with 11 males (78.6%) and 3 females (21.4%). Only one patient was immunocompromised, receiving azathioprine and hydroxychloroquine for systemic lupus erythematosus. Other reported comorbidities included diabetes mellitus (2 [14.3%]), heart disease (2 [14.3%]), tuberculosis (1 [7.1%]) and cirrhosis (1 [7.1%]) (Table 1). Most cases presented with the acute/subacute clinical form (8 [57.1%]), after a median delay of 13 weeks (range: 1–52 weeks) from symptom onset. All patients had multifocal organ involvement, with weight loss (11 [78.6%]) and fever (10 [71.4%]) being the most common presenting symptoms.

**TABLE 1:** Case series of osteoarticular, bone marrow and urogenital paracoccidioidomycosis at HCFMUSP (2006–2025).

Age (years)	Sex	Comorbidities	Clinical form	Atypical site	Involvement	Laboratory	Diagnosis	Treatment	Outcome
4	F	None	A/S	OA	Multifocal osteomyelitis	Anaemia Eosinophilia	LFN histology Serology	AMB ITC	NI
23	M	None	A/S	OA	Unifocal osteomyelitis	Anaemia Eosinophilia	LFN histology Serology	AMB ITC	Improvement
22	M	None	A/S	OA	Unifocal osteomyelitis	Eosinophilia	LFN MD and histology Serology	AMB	Death
22	F	Cirrhosis	A/S	OA	Multifocal osteomyelitis	Anaemia Eosinophilia	LFN MD, culture and histology Serology	SMX‐TMP	Improvement
21	M	None	A/S	OA	Multifocal osteomyelitis Monoarticular arthritis	Anaemia Eosinophilia	LFN histology Serology	AMB ITC	Improvement
45	M	None	C	OA	Unifocal osteomyelitis	None	CNS histology Serology	SMX‐TMP	Improvement
66	M	None	C	OA	Unifocal osteomyelitis	None	Skin MD and histology Serology	ITC	Improvement
20	M	None	A/S	OA BM	Unifocal osteomyelitis	Anaemia	BM MD and histology Serology	AMB SMX‐TMP	NI
60	M	DM Heart disease	C	OA	Multifocal osteomyelitis	Anaemia	Bone MD, culture and histology Serology	ITC	Improvement
65	F	Heart disease SLE	C	OA	Multifocal osteomyelitis Monoarticular arthritis	Anaemia	Bone histology Serology	SMX‐TMP	Improvement
55	M	None	A/S	UG	Unilateral testicle and epididymis	Anaemia Eosinophilia	Testicle MD and histology Serology	ITC	NI
62	M	None	C	UG	Unilateral testicle	None	Testicle histology Serology	ITC	Improvement
35	M	Tuberculosis	A/S	BM UG	Unilateral testicle	Anaemia Eosinophilia	BM culture and histology Serology	None	Death
61	M	DM Heart disease	C	UG	Penis	None	Penis histology Serology	KTC	Death

Abbreviations: A/S, acute/subacute; AMB, amphotericin B; BM, bone marrow; C, chronic; CNS, central nervous system; DM, diabetes mellitus; ITC, itraconazole; KTC, ketoconazole; LFN, lymph node; MD, mycological direct exam; NI, not informed; OA, osteoarticular; SLE, systemic lupus erythematosus; SMX‐TMP, sulfamethoxazole–trimethoprim; UG, urogenital.

In cases with osteoarticular involvement, local pain (4 [40.0%]), local edema (2 [20.0%]) and fistula formation (1 [10.0%]) were observed; no patient presented with local hyperemia. Unifocal osteomyelitis (5 [50.0%]) and multifocal osteomyelitis (5 [50.0%]) were the most common clinical presentations, followed by monoarthritis (2 [20.0%]). The most frequently affected sites were the rib (3 [30.0%]) and clavicle (3 [30.0%]); other sites included the knee (2 [20.0%]), vertebrae (2 [20.0%]), sternum, manubriosternal joint, ilium, tibia and scapula (1 [10.0%] each). CT and X‐ray were performed in all cases, and MRI in two cases (2 [20.0%]). Radiological findings included lytic lesions (10 [100%]), soft tissue edema (3 [30.0%]), abscess formation (1 [10.0%]), synovitis (1 [10.0%]) and joint effusion (1 [10.0%]). No periosteal reaction was observed.

In urogenital PCM, no patient presented with urinary urgency, nocturia, hematuria, pollakiuria, dysuria, or urinary retention. Cutaneous lesions were observed in two cases (2 [50.0%]), both presenting as ulcers. Local pain and local edema were reported in three (3 [75.0%]) and one (1 [25.0%]) patient, respectively. The testicle was the most commonly affected site (3 [75.0%]), all with unilateral involvement. The epididymis and penis were each involved in one case (1 [25.0%]).

Laboratory abnormalities for all atypical forms were summarized in Table 1. Diagnostic methods included mycological direct exam, culture, histopathology and serology. No patient received a post‐mortem diagnosis; however, in one case, testicular involvement was identified only at autopsy. A specific microbiological diagnosis at the atypical site was achieved in only half of the cases. In the remaining patients, diagnosis was established based on microbiological confirmation at another site combined with clinical and/or radiological findings. Serology was positive in all patients. Table 1 summarizes the diagnostic methods used.

The majority of patients required hospitalization (12 [85.7%]), whereas surgical intervention was required in a minority of cases, all of which were orchiectomies (2 [14.3%]). Antifungal therapy was administered to 13 patients (13 [92.9%]), with intravenous amphotericin B used in five cases (5 [35.7%]). Additional antifungal agents included itraconazole (7 [50.0%]), sulfamethoxazole–trimethoprim (SMX–TMP) (4 [28.6%]) and ketoconazole (1 [7.1%]). Outcome data were available for 11 patients; among them, three (27.3%) died and eight (72.7%) showed clinical improvement.

### 3.2 Systematic Review

**FIGURE 1:**
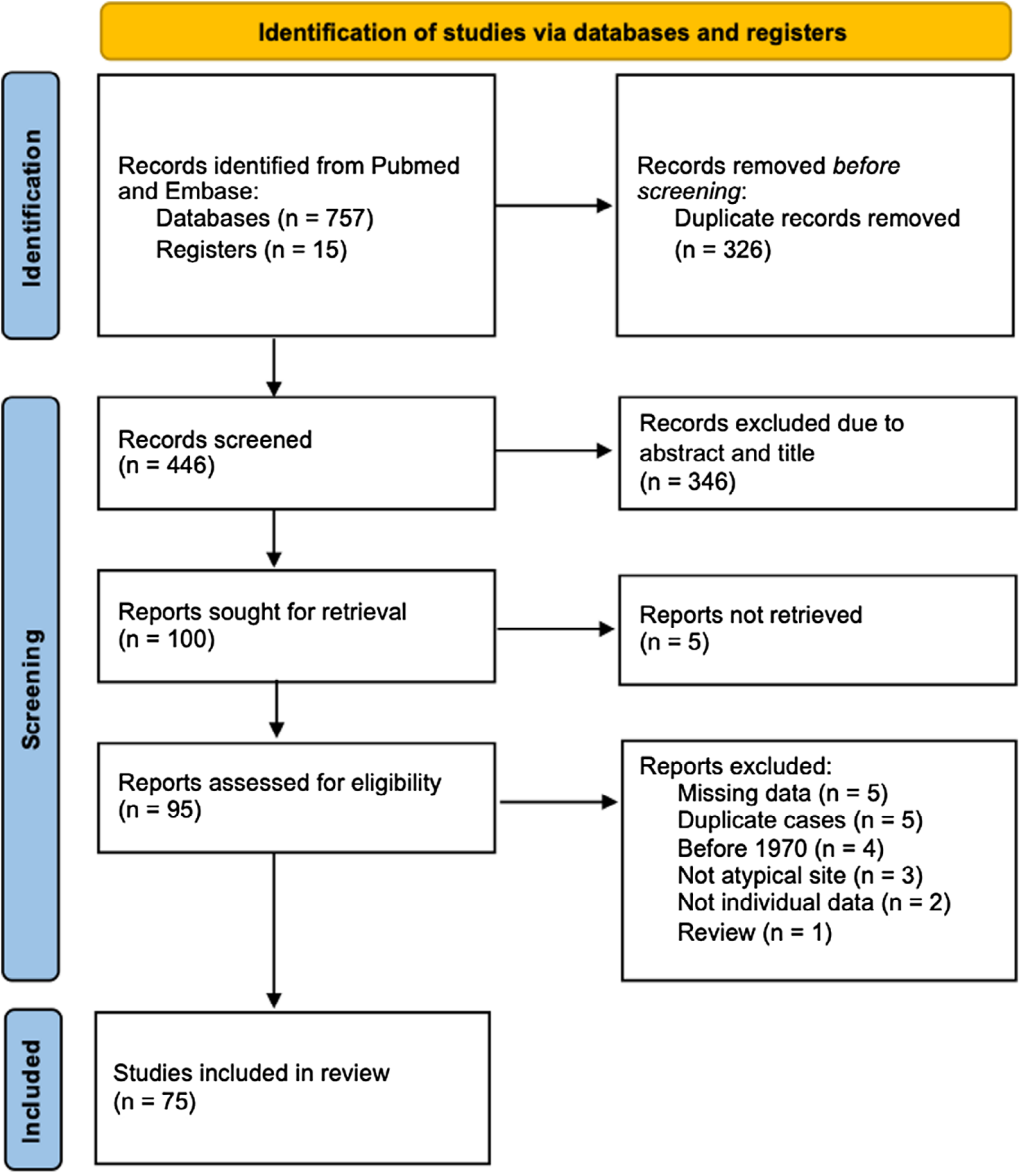
Flowchart describing the total of scientific articles obtained by the database searches for atypical paracoccidioidomycosis cases.

The final review included 75 articles published between 1970 and 2025, describing a total of 153 cases. Overlapping involvement of atypical sites was observed in five cases: one involving bone marrow, osteoarticular and urogenital systems; two involving bone marrow and osteoarticular sites; one involving bone marrow and urogenital sites; and one involving urogenital and osteoarticular sites. A flowchart illustrating the search strategy is shown in Figure 1. When combining our case series with the literature review (*n* = 167 total cases), the majority of cases were from Brazil (151 [90.4%]), followed by the United States (4 [2.4%]) and Colombia (3 [1.8%]).

### 3.3 Osteoarticular

We identified 10 cases of PCM with osteoarticular involvement over a 19‐year period (Table 1). The PubMed/MEDLINE and Embase searches identified 80 additional cases up to November 2025. The median age of cases with osteoarticular involvement was 29 years (range: 3–82), with a predominance of males (71/87 [81.6%]) and the acute/subacute clinical form (51/85 [60.0%]) (Table 2). Among patients with available data, most were previously healthy (49 [70.0%]). Among those with comorbidities (21 [30.0%]), the most frequent conditions were heart disease (6 [28.6%]), HIV infection (4 [19.0%]; median CD4 count: 94 cells/mm^3^; range: 9–291), tuberculosis (3 [14.3%]), diabetes mellitus (2 [9.5%]), autoimmune diseases (2 [9.5%]), prior solid organ transplantation (2 [9.5%]), haematological malignancies (2 [9.5%]), chronic kidney disease (1 [4.8%]) and cirrhosis (1 [4.8%]).

**TABLE 2:** Demographic, clinical, treatment, and outcome characteristics of all patients with osteoarticular (*n* = 90), bone marrow (*n* = 31), and urogenital (*n* = 52) paracoccidioidomycosis.

Variable	Osteoarticular PCM *n* (%)	Bone marrow PCM *n* (%)	Urogenital PCM *n* (%)
Median age (years)	29	18	51
Male sex	71/87 (81.6)^a^	18 (59.1)	49 (94.2)
Acute/subacute form	51/85 (60.0)^a^	31 (100)	5 (9.8)
Chronic form	34/85 (40.0)^a^	0 (0)	47 (90.4)
Arthritis	32 (35.5)	—	—
Osteomyelitis	74 (82.2)	—	—
Myositis	13 (14.4)	—	—
Tenosynovitis/bursitis	4 (4.4)	—	—
Joint effusion	13 (14.4)	—	—
Synovitis	10 (11.1)	—	—
Abscess	14 (15.5)	—	—
Soft tissue edema	34 (37.8)	—	—
Lytic lesions	67 (74.4)	—	—
Periosteal reaction	9 (10.0)	—	—
Testicular involvement	—	—	20 (38.5)
Prostate involvement	—	—	13 (25.0)
Penis involvement	—	—	12 (23.1)
Epididymis involvement	—	—	11 (21.1)
Scrotum involvement	—	—	6 (11.5)
Renal involvement	—	—	2 (3.9)
Anaemia	—	13/13 (100)^a^	—
Eosinophilia	—	7/13 (53.8)^a^	—
Pancytopenia	—	2/13 (15.4)^a^	—
Microscopy positive	19/26 (73.1)^a^	16/20 (80.0)^a^	12/13 (92.3)^a^
Culture positive	16/21 (76.2)^a^	5/7 (71.3)^a^	7/9 (77.8)^a^
Histology positive	42/42 (100)^a^	21/21 (100)^a^	38/39 (97.4)^a^
Serology positive	26/33 (78.8)^a^	9/9 (100)^a^	24/25 (96.0)^a^
Post‐mortem diagnosis	0 (0)	3 (9.7)	3 (5.9)
Amphotericin B	29/76 (38.2)^a^	10/19 (52.6)^a^	7/23 (30.4)^a^
SMX‐TMP	38/76 (50.0)^a^	6/19 (31.6)^a^	8/23 (34.8)^a^
Itraconazole	30/76 (39.5)^a^	5/19 (26.3)^a^	11/23 (47.8)^a^
Other antifungals	11/76 (14.5)^a^	0/19 (0)^a^	3/23 (13.0)^a^
Surgery	13 (14.4)	—	17 (32.7)
Improvement	53/59 (89.8)^a^	4/18 (22.2)^a^	17/25 (68.0)^a^
Death	6/59 (10.2)^a^	14/18 (77.8)^a^	8/25 (32.0)^a^

Abbreviations: PCM, paracoccidioidomycosis; SMX‐TMP, sulfamethoxazole–trimethoprim.

^a^ Number of cases for which the information was available.

Local signs and symptoms were the most frequent, particularly pain (72 [80.0%]) and edema (45 [50.0%]). Local hyperemia (19 [21.1%]) and fistula formation (10 [11.1%]) were less common. Systemic signs and symptoms were also reported, including fever (37 [41.1%]) and weight loss (32 [35.5%]). The median time from symptom onset to diagnosis was 12 weeks (range: 1–208 weeks). X‐ray, CT, MRI and ultrasound were used in 57 (63.3%), 42 (46.7%), 27 (30.0%) and 3 (3.3%) cases, respectively. The main radiological findings were lytic lesions (67 [74.4%]) and soft tissue edema (34 [37.8%]). Osteomyelitis was the most common clinical presentation (74 [82.2%]), followed by arthritis (32 [35.5%]), myositis (13 [14.4%]) and tenosynovitis/bursitis (4 [4.4%]) (Table 2). Osteoarticular involvement was unifocal in 47 patients (47 [52.2%]) and multifocal in 31 (31 [34.4%]). Among patients with joint involvement, 75.7% presented with monoarthritis, 18.2% with oligoarthritis and 6.1% with polyarthritis.

Information on the site of involvement was available for 89 patients. The most frequently affected sites were the lower limbs (58 [65.2%]), followed by the upper limbs (20 [22.5%]), skull (16 [18.0%]), ribs (13 [14.6%]), clavicles (11 [12.4%]), wrists (10 [11.2%]), vertebrae (9 [10.1%]), sternum (9 [10.1%]) and scapula (5 [5.6%]). In the lower limbs, the most commonly affected structures were the knee (18/58 [31.0%]) and the femur (13/58 [22.4%]). Most cases (65 [72.2%]) occurred in the context of multifocal involvement, whereas 25 (27.8%) presented as single‐site involvement.

A specific bone diagnosis was reported in 51 patients (51 [56.7%]). Among these, mycological direct examination and culture were positive in 19/26 (73.1%) and 16/21 (76.2%) cases, respectively. Synovial fluid analysis was reported in only three cases: one with glucose of 85 mg/dL and protein of 5.6 g/dL; another with glucose of 50 mg/dL, protein of 5.8 g/dL and a differential cell count showing 64% neutrophils; and a third with 10,100 cells/mm^3^, of which 80% were lymphocytes. Histopathological examination was performed in 42 patients (42 [46.7%]) and yielded positive results in all cases. Serological testing was reported in 33 patients (33 [36.7%]), with positive results in 26 (26/33 [78.8%]) and negative results in 7 (7/33 [21.2%]). No patient had a post‐mortem diagnosis (Table 2).

Antifungal treatment was described in 76 cases (76 [84.4%]), including amphotericin B (29 [32.2%]), itraconazole (30 [33.3%]), sulfamethoxazole–trimethoprim (SMX–TMP) (38 [42.2%]), sulfadiazine (4 [4.4%]), voriconazole (2 [2.2%]), fluconazole (1 [1.1%]) and ketoconazole (4 [4.4%]). Outcomes were available for 59 patients, of whom 53 (89.8%) showed clinical improvement and 6 (10.2%) died. Four deaths were related to PCM. Surgery was reported in 13 patients (13 [14.4%]) (Table 2): in two cases (2/13 [15.4%]), it was used exclusively for diagnostic purposes; in four cases (4/13 [30.8%]), it was performed solely for debridement or drainage of bone and/or joint involvement; and in seven cases (7/13 [53.8%]), it served both diagnostic and therapeutic purposes. A summary of systematically reviewed clinical cases of osteoarticular PCM is provided in Appendix B.

### 3.4 Bone Marrow

We identified 3 cases of PCM with bone marrow involvement over a 19‐year period (Table 1). A PubMed/MEDLINE and Embase search identified 28 additional cases up to November 2025. Most patients were male (18 [58.1%]), and the median age was 19 years (range: 3–58) (Table 2). Comorbidities were described in five patients (5 [16.1%]): four with HIV infection (only one with a reported CD4 count of 44 cells/mm^3^) and one with tuberculosis.

All cases were classified as acute/subacute PCM, with fever (24/28 [85.7%]) and weight loss (22/28 [78.6%]) being the most frequent clinical manifestations, in association with involvement of other organs of the reticuloendothelial system, such as lymph nodes, liver and spleen. The median time from symptom onset to diagnosis was 11 weeks (range: 8–20 weeks). Among patients with available laboratory data, the most relevant findings were anaemia (13/13 [100%]) and eosinophilia (7/13 [53.8%]). In contrast, pancytopenia was a rare finding, occurring in 2/13 cases (15.4%) (Table 2).

Regarding diagnosis, bone marrow biopsy was the most frequently used diagnostic method (21 [67.7%]), followed by bone marrow aspirate (20 [64.5%]). Histology was positive in all cases in which it was performed. Mycological direct examination was positive in 16/20 cases (80.0%), while culture yielded positive results in 5/7 cases (71.4%). Serological testing was used as a complementary diagnostic method in 9 patients (9 [29.0%]; titre range: 1:1–1:512). Post‐mortem diagnosis was reported in three cases (3 [9.7%]) (Table 2).

All patients with bone marrow involvement were hospitalized. Antifungal treatment was described in 19 cases (19 [61.3%]). Among these, 10 (10/19 [52.6%]) received amphotericin B, 6 (6/19 [31.6%]) sulfamethoxazole–trimethoprim (SMX–TMP), and 5 (5/19 [26.3%]) itraconazole. Considering only patients with available outcome data, the outcome was unfavourable in 14/18 cases (77.8%), with PCM being the cause of death in 13 cases (Table 2). A summary of systematically reviewed clinical cases of bone marrow PCM is provided in Appendix C.

### 3.5 Urogenital

We identified 4 cases of PCM with urogenital involvement over a 19‐year period (Table 1). A PubMed/MEDLINE and Embase search identified 48 additional cases up to November 2025. The median age of cases with urogenital involvement was 51 years (range: 15–79), with a marked predominance of males (49 [94.2%]) and the chronic clinical form (47 [90.4%]) (Table 2). Among patients with available data, most had no comorbidities (31/45 [68.9%]). Among those with comorbidities (14/45 [31.1%]), the most frequent conditions were heart disease (6 [42.9%]), tuberculosis (3 [21.4%]), HIV infection (2 [14.3%]), diabetes mellitus (1 [7.1%]), prior solid organ transplantation (1 [7.1%]) and haematological malignancy (1 [7.1%]).

Local edema (22 [42.3%]) and local pain (17 [32.7%]), often associated with genital lesions (20 [38.5%]), were the most frequent signs and symptoms of urogenital PCM. Ulceration was the most characteristic genital cutaneous lesion, present in 16/20 cases (80.0%). Other reported signs and symptoms included weight loss (18 [34.6%]), pollakiuria (7 [13.5%]), urinary retention (6 [11.5%]), dysuria (5 [9.6%]) and urinary urgency (4 [7.7%]). Fever was infrequent (3 [5.8%]). The median time from symptom onset to diagnosis was 24 weeks (range: 1–730 weeks). Most urogenital cases were associated with multifocal involvement (46 [88.5%]), whereas only six patients (6 [11.5%]) had disease restricted to the urogenital site.

Genital involvement was the most common manifestation of urogenital PCM (50 [96.1%]), followed by renal involvement (2 [3.9%]). No urinary tract involvement was reported. The testicle was the most frequently affected site (20 [38.5%]), followed by the prostate (13 [25.0%]), penis (12 [23.1%]), epididymis (11 [21.1%]) and scrotum (6 [11.5%]). Involvement of the ovary, endometrium, vulva, and cervix was reported in only one case each (1 [1.9%] each) (Table 2). Among patients with available data, unilateral disease predominated (23/29 [79.3%]), whereas bilateral involvement occurred in 6/29 cases (20.7%).

A specific urogenital diagnosis was reported in 43 patients (43 [82.7%]). Among these, mycological direct examination and culture were positive in 12/13 (92.3%) and 7/9 (77.8%) cases, respectively. Histopathological examination was performed in 39 patients (39 [75.0%]) and yielded positive results in 38/39 cases (97.4%). Serological testing was reported in 25 patients (25 [48.1%]), with positive results in 24/25 (96.0%) and negative results in 1/25 (4.0%). Post‐mortem diagnosis was reported in three patients (3 [5.8%]) (Table 2).

Antifungal treatment was described in 23 cases (23 [44.2%]), including amphotericin B (7 [13.5%]), itraconazole (11 [21.2%]), sulfamethoxazole–trimethoprim (SMX–TMP) (8 [15.4%]), ketoconazole (2 [3.8%]) and terbinafine (1 [1.9%]). A surgical procedure was performed in 17 patients (17 [32.7%]), most of whom underwent unilateral orchiectomy (8/17 [47.1%]). Outcomes were reported for 25 patients, of whom 17 (68.0%) showed clinical improvement and 8 (32.0%) died due to PCM (Table 2). A summary of systematically reviewed clinical cases of urogenital PCM is provided in Appendix D.

## 4 Discussion

In this retrospective cohort study combined with a structured systematic literature review, we analysed 167 cases of atypical PCM involving the osteoarticular, bone marrow, and urogenital systems, most of them reported from South America and predominantly from Brazil (90%). Although these extrapulmonary manifestations are rarely emphasized in consensus guidelines and major reviews, their systematic evaluation revealed consistent clinical, diagnostic, and prognostic patterns. Rather than representing isolated rare events, these atypical forms appear to be underrecognized presentations of PCM that are frequently associated with delayed diagnosis, advanced disease at presentation, and poor outcomes.

The involvement of bone and/or joint in PCM occurs due to hematogenous dissemination, and the history of trauma, preceding manifestations, should not be viewed as an inoculating factor of the fungus, but rather as a localizing or triggering factor for the establishment of the agent during the fungemic phase [7, 8]. The bone and joint lesions are often associated with disseminated disease and are frequently incidental findings [9]. While the bone lesions are generally asymptomatic, the joint lesions present with expressive manifestations, such as intense inflammatory signs and functional impairment [7, 8, 10].

Most of the published cases involved male patients, with a median age of 29 years, presenting with the acute/subacute clinical form. Osteomyelitis was the most common clinical presentation, typically characterized by local signs such as pain and edema, often accompanied by fever and weight loss. Arthritis typically appears as an extension of epiphyseal bone lesions and has been reported to occur in approximately 40% of cases [7, 8, 9], most commonly presenting as monoarthritis. PCM can affect virtually any bone, joint, or muscle; however, the most commonly affected sites were the bones, joints and muscles of the lower limbs, including hip, femur, knee, tibia, fibula and ankle, occurring in approximately two‐thirds of the cases.

Diagnosis can be made by culture or direct examination of the synovial fluid or involved tissue, as well as histopathological examination [9, 10]. The use of conventional radiography and CT has revealed well‐defined osteolytic lesions, characterized by the absence of marginal sclerosis and periosteal reaction [8, 11]. MRI findings demonstrated areas with signal intensity equal to or higher than that of muscle on T1‐weighted images, while T2‐weighted images showed reactive soft tissue edema, and post‐gadolinium sequences revealed peripheral or heterogeneous enhancement [12]. Synovial fluid findings were reported in very few cases and demonstrated a heterogeneous inflammatory profile, including both neutrophilic and lymphocytic predominance. Although limited, these findings suggest that synovial fluid analysis in osteoarticular PCM may mimic other infectious or inflammatory arthritides, reinforcing the need for microbiological and histopathological confirmation. Treatment of bone lesions can be gradual and slow, accompanied by fibrosis and neostenogenesis, with dense trabeculae [8]. For a better prognosis and complete recovery, it is essential to suspect bone or joint involvement in endemic PCM regions, as the late diagnosis may result in irreversible lesions [10]. Across published cases, antifungal therapy varied, most commonly including amphotericin B formulations, itraconazole and SMX‐TMP, sometimes combined with surgical treatment. Surgical intervention was primarily used for diagnostic purposes or for management of complications such as abscess formation and extensive bone destruction, rather than as a definitive therapeutic strategy. Unlike other fungal infections, such as those caused by *Candida* spp., where surgery may play a central role in source control, the role of surgical intervention in osteoarticular PCM appears to be limited and adjunctive [9, 13].

Bone marrow involvement in PCM is considered rare and may be underestimated [14, 15]. This involvement is strongly associated with a reduced Th1 cellular immune response and a predominance of the Th2 immune response profile [14, 16]. All published cases analysed reported acute/subacute PCM, with a predominance among younger male patients. The most frequently observed abnormalities in the bone marrow include erythropoiesis insufficiency to compensate for anaemia, leukopenia, thrombocytopenia, and an increase in eosinophilic precursors [1, 15]. Therefore, routine investigation for bone marrow involvement is warranted when severe and disseminated PCM is suspected, particularly in the presence of decreased peripheral blood cell counts [15].

The diagnosis of bone marrow involvement in PCM is established by histopathological examination and/or the demonstration of the fungus in bone marrow samples [15]. Pathological findings described in the literature included granulomatous inflammatory reaction, reticulin fibrosis and, less often, collagen fibrosis and bone marrow necrosis [17]. However, it is important to highlight the difficulties associated with this diagnosis, ranging from clinical suspicion to the performance of specific examinations and the identification of the fungus in the bone marrow, which results in a significant proportion of cases remaining undiagnosed or being diagnosed only at autopsy. The high frequency of hospitalization, intravenous antifungal therapy and mortality, as well as the rate of post‐mortem diagnoses, reinforce the severity of this form of the disease and highlight the impact that diagnostic delays can have on patient survival.

Urogenital involvement caused by *Paracoccidioides* spp. results from hematogenous dissemination of the fungus [18]. Pooled data indicate that unilateral genital involvement is the most common manifestation, with the testicles being the most frequently affected site, followed by the prostate, penis and epididymis. Only two cases of renal PCM have been reported, and no cases of urinary tract involvement were observed. In contrast with osteoarticular and bone marrow PCM, this clinical involvement was significantly more associated with middle‐aged males, smokers, and the chronic clinical form of PCM [18, 19].

This form of disease has been described as a late manifestation during PCM development, rarely appearing as the initial complaint or isolated manifestation [18, 20]. Localized edema, pain, and genital lesions, particularly ulcerated lesions, were the most frequent clinical manifestations, while fever and other systemic manifestations were less common. Post‐mortem diagnosis was reported in approximately 6% of patients, highlighting the diagnostic challenges of this disease. Treatment is recommended in accordance with established consensus guidelines [1], considering clinical, radiological and serological criteria for therapeutic success [21]. Surgical intervention was performed in approximately one‐third of patients, most of whom underwent unilateral orchiectomy. While some procedures were carried out for diagnostic purposes in the context of suspected PCM, others were performed therapeutically due to suspected malignancy or benign prostatic hyperplasia [19, 22]. This reflects both delays in diagnosis and the severity of PCM disease.

The recognition of these atypical patterns is clinically relevant. Osteoarticular PCM should be suspected in young patients with monoarthritis or osteomyelitis in endemic regions [9, 10]; bone marrow involvement should be investigated in severe acute/subacute cases presenting with anaemia and eosinophilia [14, 15, 23]; and urogenital PCM should be considered in middle‐aged men with chronic genital lesions or prostatic syndromes [18, 19, 20]. When comparing outcomes across atypical forms, bone marrow PCM exhibited markedly poor outcomes, with a mortality approximately 7.6‐fold higher than that observed in osteoarticular PCM and 2.4‐fold higher than in urogenital PCM. Clear differences were also observed in the association with clinical form: bone marrow involvement occurred exclusively in the acute/subacute presentation, whereas urogenital PCM was nine times more frequently associated with the chronic form than with the acute/subacute form. In addition, post‐mortem diagnosis was reported predominantly in bone marrow and urogenital cases, while no such reports were identified in osteoarticular involvement. The markedly elevated mortality observed in bone marrow PCM likely reflects a combination of factors, including advanced disseminated disease, diagnostic delay, and the intrinsic severity of acute/subacute presentations involving the reticuloendothelial system. Although comorbidities such as HIV infection were present in a subset of patients, they do not appear to fully explain the high mortality observed. Delayed diagnosis, including cases identified only at autopsy, further underscores the impact of limited clinical suspicion in atypical presentations. These proportional differences further reinforce that these atypical manifestations follow distinct clinical and prognostic patterns rather than representing random rare presentations of the disease. Across published cases, antifungal treatment duration in atypical PCM appeared prolonged and heterogeneous, particularly in osteoarticular involvement. Given this variability, treatment discontinuation in clinical practice should follow established criteria used for classical PCM, including sustained clinical improvement, radiological resolution or stabilization, and serological response when available [1].

This study has important limitations. The pooled analysis was based on published case reports, which are inherently subject to publication bias, heterogeneity of reporting and incomplete data. The definition of atypical involvement based on a < 5% frequency threshold was used to standardize site selection in the absence of established criteria, which may have influenced site inclusion. Diagnostic approaches and treatment strategies varied widely over decades, reflecting historical and regional differences in clinical practice. Another limitation is the short follow‐up reported in many cases. Consequently, data on long‐term outcomes were limited, precluding comparisons between different treatment strategies. Relapse was infrequently reported among atypical PCM cases; however, this finding should be interpreted with caution given the limited availability of follow‐up data and the heterogeneity in reporting across studies. These limitations preclude any meaningful comparison with relapse rates described in classical PCM and highlight the need for prospective studies with standardized follow‐up. The absence of standardized reporting across cases limited the ability to perform formal comparative or statistical analyses. Therefore, results should be interpreted as descriptive clinical patterns rather than quantitative estimates of frequency or risk. Additionally, the cases reported in the literature likely represent the most severe or unusual presentations, which may overestimate the apparent morbidity and mortality of these atypical forms.

## 5 Conclusion

To our knowledge, this is the first study to systematically characterize the clinical phenotype of atypical extrapulmonary PCM manifestations rather than describing them as isolated rare events. Most cases summarized here originated from Brazil, the epicentre of the disease, highlighting the relevance of these findings for endemic regions. The high rates of post‐mortem diagnosis, morbidity, and mortality observed across these cases likely reflect delayed recognition due to unfamiliarity with these unusual clinical presentations. Recognizing the distinct patterns of osteoarticular, bone marrow, and urogenital involvement may assist clinicians in earlier suspicion, targeted investigation and timely treatment. These findings reinforce that atypical PCM should be understood as recognizable clinical entities within the disease spectrum rather than anecdotal presentations.

## Author Contributions

**Mariane Cappellini Stricagnolo:** data curation, methodology, formal analysis, writing – original draft. **Marcello Mihailenko Chaves Magri:** conceptualization, methodology, data curation, supervision, writing – review and editing. **Mônica Scarpelli Martinelli Vidal:** data curation. **Adriana Satie Gonçalves Kono Magri:** data curation, supervision, writing – review and editing. **Gil Benard:** data curation. **Tiago Alexandre Cocio:** methodology, conceptualization. **Vítor Falcão de Oliveira:** data curation, writing – review and editing, supervision. **Wdson Luis Lima Kruschewsky:** conceptualization, methodology, formal analysis, data curation, writing – original draft, writing – review and editing.

## Funding

The authors have nothing to report.

## Conflicts of Interest

The authors declare no conflicts of interest.

## Supporting information


**Data S1:** PRISMA 2020 checklist.

## Data Availability

The data that support the findings of this study are available on request from the corresponding author. The data are not publicly available due to privacy or ethical restrictions.

## Appendix A Strategies of the Search in Each Database

### PubMed/MEDLINE

**Table:**

Strategy
((“Urogenital”[Mesh]) OR (“Urinary Tract”[Mesh]) OR (“Urological”[Mesh]) OR (“Genital”[Mesh]) OR (“Testicular”[Mesh]) OR (“Prostate”[Mesh]) OR (“Kidney”[Mesh]) OR (“Bone marrow”[Mesh]) OR (“Bone”[Mesh]) OR (“Joint”[Mesh]) OR (“Osteoarticular”[Mesh]) OR (“Osteomyelitis”[Mesh]) OR (“Arthritis”[Mesh]) OR (“Tenosynovitis”[Mesh]) OR (“Bursitis”[Mesh]) OR (“Myositis”[Mesh]) OR (“Spondylodiscitis”[Mesh]) OR (“Vertebral”[Mesh]) AND ((“Paracoccidioidomycosis”[Mesh]) OR (“Paracoccidioidal”) OR (“Paracoccidioides”) OR (“South American Blastomycosis”)))

### EMBASE

**Table:**

Strategy
(‘urogenital’/exp. OR ‘urinary tract’/exp. OR ‘urological’/exp. OR ‘genital’/exp. OR ‘testicular’/exp. OR ‘prostate’/exp. OR ‘kidney’/exp. OR ‘bone marrow’/exp. OR ‘bone’/exp. OR ‘joint’/exp. OR ‘osteoarticular’/exp. OR ‘osteomyelitis’/exp. OR ‘arthritis’/exp. OR ‘tenosynovitis’/exp. OR ‘bursitis’/exp. OR ‘myositis’/exp. OR ‘spondylodiscitis’/exp. OR ‘vertebral’/exp) AND (‘paracoccidioidomycosis’/exp. OR ‘paracoccidioidal’ OR ‘paracoccidioides’ OR ‘south american blastomycosis’)

## Appendix B Summary of Systematically Reviewed Clinical Cases of Osteoarticular Paracoccidioidomycosis

**Table:**

Reference	Country	Age	Sex	Clinical form	Involvement	Site	Surgery	Treatment	Outcome
Krivoy, 1978 [24]	Venezuela	27	F	C	Osteomyelitis	Unifocal	Yes	SMX‐TMP	NI
Chikamori, 1984 [25]	Japan	34	F	A/S	Osteomyelitis	Multifocal	None	AMB KTC	Improvement
Rosario Filho, 1985 [26]	Brazil	3	M	A/S	Osteomyelitis	Multifocal	None	AMB SMX‐TMP	Improvement
Rosario Filho, 1985 [26]	Brazil	5	M	A/S	Osteomyelitis	Unifocal	None	SMX‐TMP KTC	Improvement
Lazow, 1990 [27]	USA	59	M	C	Osteomyelitis	Unifocal	Yes	AMB KTC	Improvement
Shikanai, 1992 [23]	Brazil	27	M	A/S	Osteomyelitis	Multifocal	None	AMB ITC SMX‐TMP	Death
Campos, 1992 [28]	Brazil	6	M	A/S	Osteomyelitis	Multifocal	None	SMX‐TMP	Improvement
Benard, 1995 [29]	Brazil	8	F	A/S	Osteomyelitis Arthritis	Unifocal Oligoarticular	None	AMB SMX‐TMP	Improvement
Benard, 1995 [29]	Brazil	10	M	A/S	Osteomyelitis	Unifocal	None	AMB SMX‐TMP	Improvement
Benard, 1995 [29]	Brazil	7	F	A/S	Osteomyelitis	Multifocal	None	AMB	Death
Severo, 1996 [30]	Brazil	NI	M	NI	Osteomyelitis	Unifocal	NI	NI	NI
Severo, 1996 [30]	Brazil	NI	M	NI	Osteomyelitis	Unifocal	NI	NI	NI
Severo, 1996 [30]	Brazil	NI	M	NI	Osteomyelitis	Unifocal	NI	NI	NI
Severo, 1996 [30]	Brazil	47	M	NI	Osteomyelitis	Unifocal	NI	NI	NI
Severo, 1996 [30]	Brazil	31	M	NI	Osteomyelitis	Unifocal	NI	NI	NI
Amstalden, 1996 [9]	Brazil	29	M	A/S	Arthritis	Monoarticular	None	NI	NI
Amstalden, 1996 [9]	Brazil	49	M	C	Arthritis	Monoarticular	Yes	SMX‐TMP	NI
Amstalden, 1996 [9]	Brazil	45	M	C	Osteomyelitis Arthritis	Multifocal Monoarticular	None	SMX‐TMP	NI
Amstalden, 1996 [9]	Brazil	46	M	C	Osteomyelitis Arthritis	Unifocal Monoarticular	None	SMX‐TMP	Improvement
Amstalden, 1996 [9]	Brazil	37	M	C	Arthritis Myositis	Unifocal Monoarticular	None	AMB SMX‐TMP	Improvement
Amstalden, 1996 [9]	Brazil	29	M	A/S	Arthritis	Monoarticular	None	SMX‐TMP	Improvement
Amstalden, 1996 [9]	Brazil	39	M	C	Osteomyelitis Arthritis	Multifocal Monoarticular	None	SMX‐TMP	Improvement
Amstalden, 1996 [9]	Brazil	19	M	A/S	Osteomyelitis Arthritis	Unifocal Monoarticular	None	SMX‐TMP	Improvement
Amstalden, 1996 [9]	Brazil	9	M	A/S	Osteomyelitis	Unifocal	None	SMX‐TMP	Improvement
Fulciniti, 1996 [31]	Italy	60	M	C	Osteomyelitis Arthritis	Unifocal Monoarticular	None	ITC	Improvement
Doria, 1997 [32]	Brazil	4	M	A/S	Osteomyelitis	Multifocal	NI	NI	NI
David, 1997 [33]	Brazil	31	M	C	Osteomyelitis	Unifocal	Yes	Sulfadiazine	Improvement
Silvestre, 1997 [10]	Brazil	46	F	C	Arthritis	Monoarticular	None	SMX‐TMP KTC	Improvement
Miranda Aires, 1997 [34]	Brazil	18	M	A/S	Osteomyelitis	Unifocal	Yes	AMB ITC	NI
Borgia, 2000 [35]	Italy	61	M	C	Osteomyelitis	Unifocal	None	ITC	Improvement
Scheinberg, 2001 [36]	Brazil	52	M	C	Arthritis	Oligoarticular	None	Sulfadiazine	Improvement
Nogueira, 2001 [37]	Brazil	7	F	A/S	Osteomyelitis	Multifocal	Yes	AMB	Improvement
Lambertucci, 2002 [38]	Brazil	20	M	A/S	Osteomyelitis	Unifocal	None	SMX‐TMP	Improvement
Picado, 2003 [39]	Brazil	21	F	A/S	Osteomyelitis	Unifocal	None	SMX‐TMP	Improvement
Cassone, 2005 [40]	Brazil	75	M	C	Osteomyelitis	Unifocal	None	ITC	Improvement
Tresoldi, 2006 [41]	Brazil	6	M	A/S	Osteomyelitis	Multifocal	None	AMB SMX‐TMP	Improvement
Castro, 2006 [42]	Brazil	32	M	C	Osteomyelitis	Multifocal	None	AMB ITC	Death
Valera, 2008 [43]	Brazil	10	M	A/S	Osteomyelitis	Unifocal	None	NI	NI
Neves, 2008 [44]	Brazil	12	M	A/S	Myositis	Multifocal	Yes	AMB ITC	NI
Navarro, 2008 [45]	Colombia	12	M	A/S	Osteomyelitis Arthritis Tenosynovitis/Bursitis	Multifocal Oligoarticular	None	AMB ITC SMX‐TMP	NI
Freitas, 2010 [46]	Brazil	47	M	C	Osteomyelitis	Unifocal	None	AMB ITC SMX‐TMP	Improvement
Pereira, 2011 [47]	Brazil	20	M	A/S	Osteomyelitis	Multifocal	None	AMB SMX‐TMP	Improvement
Bayerl, 2012 [48]	Brazil	59	M	C	Osteomyelitis	Unifocal	None	AMB SMX‐TMP	NI
Bonilla‐Abadía, 2012 [49]	Colombia	82	M	A/S	Arthritis	Monoarticular	Yes	ITC	NI
Correa‐de‐Castro, 2012 [50]	Brazil	47	M	A/S	Osteomyelitis	Unifocal	None	SMX‐TMP	Improvement
Marchiori, 2012 [51]	Brazil	24	M	A/S	Osteomyelitis Arthritis	Unifocal Monoarticular	None	NI	NI
Von Glehn, 2012 [52]	Brazil	63	M	C	Arthritis Tenosynovitis/Bursitis	Multifocal Poliarticular	Yes	ITC SMX‐TMP	Improvement
Michelan, 2013 [53]	Brazil	11	M	A/S	Osteomyelitis Arthritis	Unifocal Monoarticular	Yes	FLC	NI
Safe, 2014 [54]	Brazil	28	M	A/S	Arthritis	Monoarticular	Yes	AMB	Death
Marques, 2014 [55]	Brazil	71	M	A/S	Osteomyelitis	Unifocal	None	AMB SMX‐TMP	Improvement
Lima Júnior, 2014 [8]	Brazil	24	M	A/S	Osteomyelitis	Unifocal	NI	NI	NI
Lima Júnior, 2014 [8]	Brazil	33	M	C	Osteomyelitis	Multifocal	NI	NI	NI
Lima Júnior, 2014 [8]	Brazil	16	M	A/S	Osteomyelitis Arthritis	Multifocal Monoarticular	NI	NI	NI
Lima Júnior, 2014 [8]	Brazil	10	F	A/S	Osteomyelitis	Unifocal	NI	NI	NI
Mendonça, 2014 [56]	Brazil	55	M	C	Arthritis Tenosynovitis/Bursitis	Multifocal Oligoarticular	None	SMX‐TMP	Improvement
Savarese, 2015 [12]^a^	Brazil	30^b^	8 M 3 F	4 C 7 A/S	7 Osteomyelitis 7 Arthritis 8 Myositis	6 Unifocal 1 Multifocal 5 Monoarticular 2 Oligoarticular	1 Yes 10 None	6 ITC 3 SMX‐TMP 2 Sulfadiazine	10 Improvement
Alvarenga, 2016 [57]	Brazil	68	M	C	Osteomyelitis Myositis	Multifocal	None	ITC SMX‐TMP	Improvement
Lambaré, 2017 [58]	Paraguay	73	M	C	Osteomyelitis	Unifocal	None	AMB SMX‐TMP	NI
Ghani, 2018 [59]	USA	35	M	A/S	Osteomyelitis Arthritis	Multifocal Monoarticular	None	ITC	NI
Peçanha‐Pietrobom, 2019 [60]	Brazil	53	M	A/S	Osteomyelitis Arthritis	Multifocal Monoarticular	None	AMB SMX‐TMP	Improvement
Franco, 2019 [61]	Brazil	8	F	A/S	Osteomyelitis	Multifocal	None	ITC	NI
Kruschewsky, 2021 [62]	Brazil	26	M	A/S	Osteomyelitis	Unifocal	None	ITC	Improvement
Pereira, 2022 [63]	Brazil	59	F	C	Osteomyelitis Myositis	Multifocal	None	SMX‐TMP VOR	NI
Barguil Macedo, 2022 [64]	Brazil	65	F	A/S	Arthritis	Monoarticular	None	VOR	Improvement
Berbeo‐Velásquez, 2022 [65]	Colombia	59	M	C	Osteomyelitis	Unifocal	None	AMB ITC	NI
Maiolo, 2024 [66]	Brazil	18	M	A/S	Osteomyelitis	Multifocal Poliarticular	None	AMB	NI
Appenzeller, 2024 [67]	Brazil	41	F	C	Osteomyelitis	Multifocal	None	ITC	Improvement
Viana, 2025 [68]	Brazil	47	M	C	Osteomyelitis	Unifocal	None	ITC	Improvement
Duarte‐Neto, 2025 [69]	Brazil	67	M	C	Osteomyelitis	Unifocal	None	None	Death
Moral, 2021 [70]	Spain	29	F	A/S	Osteomyelitis Tenosynovitis/Bursitis Myositis	Unifocal	Yes	AMB ITC	Improvement
Kruschewsky, 2026	Brazil	4	F	A/S	Osteomyelitis	Multifocal	None	AMB ITC	NI
Kruschewsky, 2026	Brazil	23	M	A/S	Osteomyelitis	Unifocal	None	AMB ITC	Improvement
Kruschewsky, 2026	Brazil	22	M	A/S	Osteomyelitis	Unifocal	None	AMB	Death
Kruschewsky, 2026	Brazil	22	F	A/S	Osteomyelitis	Multifocal	None	SMX‐TMP	Improvement
Kruschewsky, 2026	Brazil	21	M	A/S	Osteomyelitis Arthritis	Multifocal Monoarticular	None	AMB ITC	Improvement
Kruschewsky, 2026	Brazil	45	M	C	Osteomyelitis	Unifocal	None	SMX‐TMP	Improvement
Kruschewsky, 2026	Brazil	66	M	C	Osteomyelitis	Unifocal	None	ITC	Improvement
Kruschewsky, 2026	Brazil	20	M	A/S	Osteomyelitis	Unifocal	None	AMB SMX‐TMP	NI
Kruschewsky, 2026	Brazil	60	M	C	Osteomyelitis	Multifocal	None	ITC	Improvement
Kruschewsky, 2026	Brazil	65	F	C	Osteomyelitis Arthritis	Multifocal Monoarticular	None	SMX‐TMP	Improvement

Abbreviations: A/S, acute/subacute; AMB, amphotericin B; C, chronic; F, female; FLC, fluconazole; ITC, itraconazole; KTC, ketoconazole; M, male; NI, not informed; SMX‐TMP, sulfamethoxazole‐trimethoprim; USA, United States of America; VOR, voriconazole,

^a^ Case series with eleven cases.

^b^ Median age.

## Appendix C Summary of Systematically Reviewed Clinical Cases of Bone Marrow Paracoccidioidomycosis

**Table:**

Reference	Country	Age	Sex	Clinical form	Laboratory	Diagnosis	Treatment	Outcome
Pedro, 1989 [71]	Brazil	37	M	A/S	A + P	MD	AMB	Death
Shikanai, 1992 [23]	Brazil	27	M	A/S	A + E	MD Culture Serology	AMB ITC SMX‐TMP	Death
Shikanai, 1992 [23]	Brazil	18	M	A/S	A + E	MD Serology	AMB	NI
Shikanai, 1992 [23]	Brazil	18	M	A/S	A + E	MD Serology	AMB	NI
Ozaki, 1996 [15]	Brazil	33	M	A/S	A	MD Culture	SMX‐TMP	Death
Resende, 2006 [17]	Brazil	19	M	A/S	NI	Histology	NI	NI
Resende, 2006 [17]	Brazil	58	F	A/S	NI	Histology	NI	NI
Resende, 2006 [17]	Brazil	17	M	A/S	NI	MD Histology	NI	NI
Resende, 2006 [17]	Brazil	40	M	A/S	NI	MD Histology	NI	NI
Resende, 2006 [17]	Brazil	12	F	A/S	NI	MD Histology	NI	NI
Resende, 2006 [17]	Brazil	17	F	A/S	NI	MD Histology	NI	NI
Resende, 2006 [17]	Brazil	19	F	A/S	NI	MD Histology	NI	NI
Resende, 2006 [17]	Brazil	7	F	A/S	NI	MD	NI	NI
Resende, 2006 [17]	Brazil	38	F	A/S	NI	Histology	NI	NI
Navarro, 2008 [45]	Colombia	12	M	A/S	A	MD Culture Histology Serology	AMB ITC SMX‐TMP	NI
Resende, 2009 [72]	Brazil	4	F	A/S	NI	Autopsy	NI	Death
Resende, 2009 [72]	Brazil	6	M	A/S	NI	Histology	NI	Death
Resende, 2009 [72]	Brazil	21	M	A/S	NI	MD	NI	Death
Resende, 2009 [72]	Brazil	9	F	A/S	NI	Histology	NI	Death
Resende, 2009 [72]	Brazil	18	F	A/S	NI	Histology	NI	Death
Resende, 2009 [72]	Brazil	19	F	A/S	NI	Histology	NI	Death
Resende, 2009 [72]	Brazil	23	F	A/S	NI	Histology	NI	Death
Resende, 2009 [72]	Brazil	21	M	A/S	NI	Histology	NI	Death
Pereira, 2010 [14]	Brazil	12	F	A/S	A	Histology Serology	ITC	Improvement
Safe, 2014 [54]	Brazil	28	M	A/S	NI	Autopsy	AMB	Death
Macedo, 2016 [73]	Brazil	18	M	A/S	A + E	MD Culture Histology Serology	AMB SMX‐TMP	Improvement
Almeida, 2017 [74]	Brazil	30	M	A/S	A + *P*	Autopsy	None	Death
Rodriguez‐Portilla, 2021 [75]	Peru	3	F	A/S	A + E	MD Histology	AMB ITC SMX‐TMP	Improvement
Kruschewsky, 2026	Brazil	35	M	A/S	A + E	Culture Histology Serology	None	Death
Kruschewsky, 2026	Brazil	20	M	A/S	A	MD Histology Serology	AMB SMX‐TMP	NI
Kruschewsky, 2026	Brazil	23	M	A/S	A + E	Histology Serology	AMB ITC	Improvement

Abbreviations: A, anaemia; A/S, acute/subacute; AMB, amphotericin B; C, chronic; E, eosinophilia; F, female; ITC, itraconazole; M, male; MD, mycological direct exam; NI, not informed; P, pancytopenia; SMX‐TMP, sulfamethoxazole‐trimethoprim.

## Appendix D Summary of Systematically Reviewed Clinical Cases of Urogenital Paracoccidioidomycosis

**Table:**

Reference	Country	Age	Sex	Clinical form	Involvement	Laterality	Surgery	Treatment	Outcome
Murray, 1974 [76]	USA	51	M	C	Testicle	Unilateral	None	AMB	Improvement
Frias, 1979 [77]	Brazil	54	M	C	Testicle Epididymis	Unilateral	Yes	AMB	Improvement
Cechella, 1982 [18]	Brazil	48	M	C	Testicle Epididymis	Unilateral	Yes	NI	NI
Cechella, 1982 [18]	Brazil	44	M	C	Testicle Prostate	Unilateral	None	NI	NI
Cechella, 1982 [18]	Brazil	50	M	C	Testicle Epididymis	Unilateral	Yes	NI	NI
Cechella, 1982 [18]	Brazil	57	M	C	Testicle Epididymis	Unilateral	None	NI	NI
Cechella, 1982 [18]	Brazil	33	M	C	Testicle Epididymis	Unilateral	Yes	NI	NI
Cechella, 1982 [18]	Brazil	51	M	C	Testicle Epididymis Prostate	Unilateral	None	NI	NI
Cechella, 1982 [18]	Brazil	72	M	C	Prostate	NA	None	NI	NI
Campos, 1986 [78]	Brazil	57	F	C	Ovary Endometrium	Unilateral	Yes	NI	NI
Melo, 1992 [79]	Brazil	71	M	C	Prostate	NA	Yes	KTC	NI
Tomimori‐Yamashita, 1997 [80]	Brazil	49	M	C	Scrotum	Unilateral	None	SMX‐TMP	Improvement
Severo, 2000 [19]	Brazil	30	M	C	Prostate	NA	None	NI	NI
Severo, 2000 [19]	Brazil	67	M	C	Testicle	Unilateral	Yes	NI	NI
Severo, 2000 [19]	Brazil	34	M	C	Testicle	Unilateral	None	NI	NI
Severo, 2000 [19]	Brazil	66	M	C	Testicle	Unilateral	None	NI	NI
Severo, 2000 [19]	Brazil	38	M	C	Testicle	Unilateral	None	NI	NI
Severo, 2000 [19]	Brazil	52	M	C	Penis	NA	None	NI	NI
Severo, 2000 [19]	Brazil	47	M	C	Epididymis	Bilateral	None	NI	NI
Severo, 2000 [19]	Brazil	34	M	C	Penis	NA	None	NI	NI
Severo, 2000 [19]	Brazil	45	M	C	Testicle Epididymis Scrotum	Unilateral	Yes	NI	NI
Severo, 2000 [19]	Brazil	50	M	C	Prostate	NA	None	NI	NI
Severo, 2000 [19]	Brazil	34	M	C	Testicle Penis	Unilateral	None	NI	NI
Ollague, 2000 [81]	Ecuador	63	M	C	Scrotum	Bilateral	None	SMX‐TMP Terbinafine	Improvement
Sheyn, 2000 [82]	USA	27	F	C	Cervix	NA	None	None	NI
Pereira, 2003 [83]	Brazil	48	M	C	Scrotum	Unilateral	None	AMB SMX‐TMP	NI
Lopes, 2009 [84]	Brazil	54	M	C	Prostate	NA	None	ITC SMX‐TMP	Improvement
Dias, 2010 [85]	Brazil	56	M	C	Penis	NA	None	SMX‐TMP	Improvement
Dias, 2010 [85]	Brazil	59	M	C	Testicle Prostate	Unilateral	Yes	ITC	Improvement
Marques, 2012 [20]	Brazil	15	F	A/S	Vulva	NA	None	NI	Death
Marques, 2012 [20]	Brazil	47	M	C	Penis	NA	None	NI	Death
Marques, 2012 [20]	Brazil	38	M	C	Penis	NA	None	ITC	Improvement
Marques, 2012 [20]	Brazil	51	M	C	Penis	NA	None	ITC	Improvement
Marques, 2012 [20]	Brazil	60	M	C	Penis	NA	None	ITC	Improvement
Marques, 2012 [20]	Brazil	40	M	C	Penis	NA	None	SMX‐TMP	Improvement
Arruda, 2013 [86]	Brazil	79	M	C	Prostate	NA	None	ITC	Death
Safe, 2014 [54]	Brazil	28	M	A/S	Kidney	Bilateral	Yes	AMB	Death
Vignolles, 2014 [87]	Argentina	74	M	C	Penis	NA	None	ITC	Improvement
Almeida, 2017 [74]	Brazil	30	M	A/S	Kidney	Bilateral	None	None	Death
Parente, 2018 [88]	Brazil	42	M	C	Scrotum	Bilateral	Yes	AMB SMX‐TMP	Improvement
Junior, 2020 [89]	Brazil	63	M	C	Testicle Epididymis	Unilateral	Yes	AMB SMX‐TMP	Improvement
Passarin, 2020 [90]	Brazil	61	M	C	Prostate	NA	None	AMB ITC	Improvement
Junior, 2021 [22]	Brazil	57	M	C	Prostate	NA	Yes	NI	NI
Junior, 2021 [22]	Brazil	52	M	C	Prostate	NA	Yes	NI	NI
Falcão, 2021 [91]	Brazil	45	M	C	Penis	NA	None	NI	NI
Souza, 2023 [92]	Brazil	73	M	C	Scrotum	Bilateral	None	ITC	Improvement
Khouri, 2025 [93]	Brazil	53	M	C	Testicle Epididymis	Unilateral	Yes	NI	NI
Duarte‐Neto, 2025 [69]	Brazil	67	M	C	Prostate	NA	None	None	Death
Kruschewsky, 2026	Brazil	55	M	A/S	Testicle Epididymis	Unilateral	Yes	ITC	NI
Kruschewsky, 2026	Brazil	62	M	C	Testicle	Unilateral	Yes	ITC	Improvement
Kruschewsky, 2026	Brazil	35	M	A/S	Testicle	Unilateral	None	None	Death
Kruschewsky, 2026	Brazil	61	M	C	Penis	NA	None	KTC	Death

Abbreviations: A/S, acute/subacute; AMB, amphotericin B; C, chronic; F, female; ITC, itraconazole; KTC, ketoconazole; M, male; NA, not applicable; NI, not informed; SMX‐TMP, sulfamethoxazole‐trimethoprim; USA, United States of America.
